# Maximum-Consistency Extension of Combined Weighted Method for Outlier-Robust Acoustic TDOA Localization

**DOI:** 10.3390/s26185791

**Published:** 2026-09-12

**Authors:** István Halász, Rozália Lakner, Gergely Zachár, Gyula Simon

**Affiliations:** 1 Alba Regia Faculty, Óbuda University, H-8000 Székesfehérvár, Hungary; halasz.istvan@amk.uni-obuda.hu (I.H.); lakner.rozalia@amk.uni-obuda.hu (R.L.); 2 Institute for Software Integrated Systems, Vanderbilt University, Nashville, TN 37212, USA; gergely.zachar@vanderbilt.edu

**Keywords:** time difference of arrival (TDOA), Cramér–Rao lower bound (CRLB), position estimation, error resilience, sensor fusion

## Abstract

Time difference of arrival (TDOA) measurements and corresponding hyperbolic localization techniques are widely utilized across diverse domains, including pedestrian tracking, wildlife monitoring, autonomous vehicle navigation, underwater positioning, and defense applications. The combined weighted (COM-W) method and its enhanced version (E-COM-W) provide location estimates in closed form in two dimensions. These methods are attractive candidates for hyperbolic localization problems because they offer a low, deterministic computational cost; require a minimal number of sensors; and incorporate geometric dilution of precision (GDOP) directly into the location estimate. While the accuracy of the location estimate approaches the Cramér–Rao lower bound (CRLB) under low-noise conditions, it degrades significantly in the presence of higher measurement errors that are common in practice. This paper elucidates the mechanisms driving this suboptimal behavior and proposes a robust method, designated as R-COM-W. The proposed algorithm not only rectifies the performance degradation of both COM-W and E-COM-W in high-noise environments but also ensures resilience against erroneous measurements, such as those caused by non-line-of-sight (NLOS) propagation. We extend this methodology to three dimensions and demonstrate the superior performance of R-COM-W using both numerical simulations and experimental measurements.

## 1. Introduction

Modern Internet of Things (IoT) applications rely heavily on localization services to track diverse targets such as autonomous vehicles [1,2], unmanned aerial vehicles (UAVs) [3,4], various assets [5], people [6,7], and wildlife [8,9]. While target localization can utilize various signal metrics, including received signal strength (RSS), angle of arrival (AOA), time of arrival (TOA), time difference of arrival (TDOA), phase of arrival (POA), round trip time (RTT), and channel state information (CSI), this paper focuses specifically on TDOA methods. TDOA systems are highly important in tracking and localization because they do not require the cooperation of the target device, enabling adoption in multiple domains, with prominent applications in sound-based systems [10,11], radio-based systems [12,13], and geolocation [14].

In a typical TDOA localization system, a signal is emitted by the target from an unknown location. This signal is detected by a number of sensors, deployed at known positions, and the time differences between the detections are utilized to determine the source’s location. Alternatively, beacons may be deployed in known positions while the target device measures the time differences between received signals. In either case, the measurements lead to the same hyperbolic localization problem [15,16]. For the sake of simplicity, we will focus our discussion on the typical case, where multiple sensors provide the TDOA measurements.

Hyperbolic localization is a nonlinear optimization problem that is often solved by iterative methods [17], although closed-form solutions also exist for configurations involving a small number of sensors [15,18,19]. Alternative approaches include consensus functions [11,12], particle filters [20], and Hough-transform-based methods [20,21]. Under small, approximately Gaussian measurement errors, these methods generally provide satisfactory estimates; however, a small number of gross errors caused by non-line-of-sight (NLOS) propagation, reflections, synchronization faults, or malfunctioning sensors can introduce substantial bias.

The precision of a position estimate depends on both measurement accuracy and sensor geometry, the latter of which is commonly characterized by the geometric dilution of precision (GDOP) [22]. The Cramér–Rao lower bound (CRLB) provides a lower bound on the covariance of any unbiased estimator by incorporating measurement-noise statistics, true target position, and sensor geometry [23]. Consequently, CRLB-derived metrics can effectively identify favorable geometric configurations, such as in CRLB-driven sensor-selection algorithms operating under LOS and NLOS conditions [24]. While such weighting primarily targets statistical efficiency and favorable geometric conditioning, it alone cannot prevent severely corrupted measurements from dominating the estimate.

Weighted estimation techniques are widely employed to improve localization robustness. Residual-based methods replace the quadratic loss with redescending or bounded-influence losses, such as Geman–McClure, Welsch, Huber, or correntropy losses, and solve the resulting problem using iteratively reweighted least squares or half-quadratic optimization [25,26,27,28]. In these approaches, measurements are dynamically assigned continuous weights based on the residuals of the current position estimate; consequently, performance inherently relies on solver convergence and proper hyperparameter tuning. Instead of relying on residual weights, another class of robust methods relies on prior uncertainty bounds. For instance, worst-case TDOA formulations apply convex relaxations assuming bounded NLOS errors [29], while robust weighted TOA schemes exploit LOS/NLOS classification and noise statistics to attenuate corrupted signals [30]. These methods remain dependent on accurate prior error bounds and channel state information.

Alternatively, hard measurement selection can be used instead of soft downweighting. Geometrical–statistical methods evaluate the Mahalanobis distance of individual or grouped TDOA measurements relative to the feasible TDOA set, iteratively pruning statistically inconsistent observations [31]. Similarly, full-set TDOA techniques identify an outlier-free subset via greedy search and random sample consensus (RANSAC) procedures prior to covariance-weighted localization [32].

The combined weighted (COM-W) method [33] provides an attractive closed-form solution for a practically relevant yet constrained localization problem. In its original formulation, it estimates a two-dimensional target position using sensors deployed in three-dimensional space. COM-W first generates preliminary position estimates from minimal sensor subsets and subsequently combines them using reciprocal CRLB-derived weights. Because the CRLB encapsulates both measurement noise statistics and geometric conditioning, preliminary estimates originating from poorly configured sensor subsets exert less influence on the final solution.

A numerical vulnerability in the calculation and selection of preliminary COM-W estimates was recently identified and resolved by the extended COM-W (E-COM-W) method [34]. Building on these developments, the present study extends both COM-W and E-COM-W—collectively denoted as (E-)COM-W—and introduces the robust COM-W (R-COM-W) method. In contrast to the robust weighted approaches discussed above, R-COM-W explicitly decouples robust hypothesis selection from statistical-efficiency weighting. It first computes closed-form position hypotheses from all four-sensor subsets and evaluates each against the full active-sensor set via a pairwise consistency criterion. It then filters the hypotheses, retaining only those that achieve maximum consistency, and fuses the surviving candidates using reciprocal CRLB-derived weights. Consequently, the CRLB weights do not serve as residual-dependent outlier downweights; instead, robustness is enforced by the preceding maximum-consistency gate, while the subsequent weighting optimizes for the geometric and statistical quality of the retained subset estimates.

The contributions of this paper are summarized as follows:

- The two-dimensional localization framework of (E-)COM-W is extended to three-dimensional target localization.
- We demonstrate that (E-)COM-W achieves CRLB-benchmarked performance under low-measurement-noise conditions. However, as the noise level increases, its estimation performance degrades. Crucially, the threshold at which this deterioration occurs aligns with typical real-world measurement noise levels, highlighting the practical significance of this limitation. We elucidate the underlying mechanisms responsible for this performance degradation.
- We introduce the R-COM-W method, which integrates exhaustive four-sensor hypothesis generation, maximum-consistency subset selection, and reciprocal-CRLB weighting. We demonstrate that R-COM-W effectively resolves the performance degradation observed in (E-)COM-W.
- Another key advantage of R-COM-W is its resilience against outlier measurements stemming from non-line-of-sight (NLOS) propagation, multipath reflections, or sensor faults. The efficacy of R-COM-W is demonstrated through both numerical simulations and real-world experiments.

The structure of the paper is as follows: In Section 2, the (E-)COM-W methods are revised and extended to 3D. Their problematic behavior is illustrated, and the reason is explained. The new R-COM-W method is proposed in Section 3. In Section 4, simulations and measurements will be used to demonstrate the performance of the proposed method. Section 5 concludes the paper.

## 2. Combined Weighted Methods for TDOA-Based Localization

This section briefly reviews the (E-)COM-W localization framework, following [33,34]. First, the fundamental TDOA localization problem is formulated. Subsequently, the elementary position estimation step of (E-)COM-W is outlined, followed by an overview of the combination scheme. The consensus function is then formally defined. Finally, the mechanism causing the performance degradation of the conventional (E-)COM-W method is illustrated and analyzed.

### 2.1. The TDOA Localization Problem

**Figure 1:**
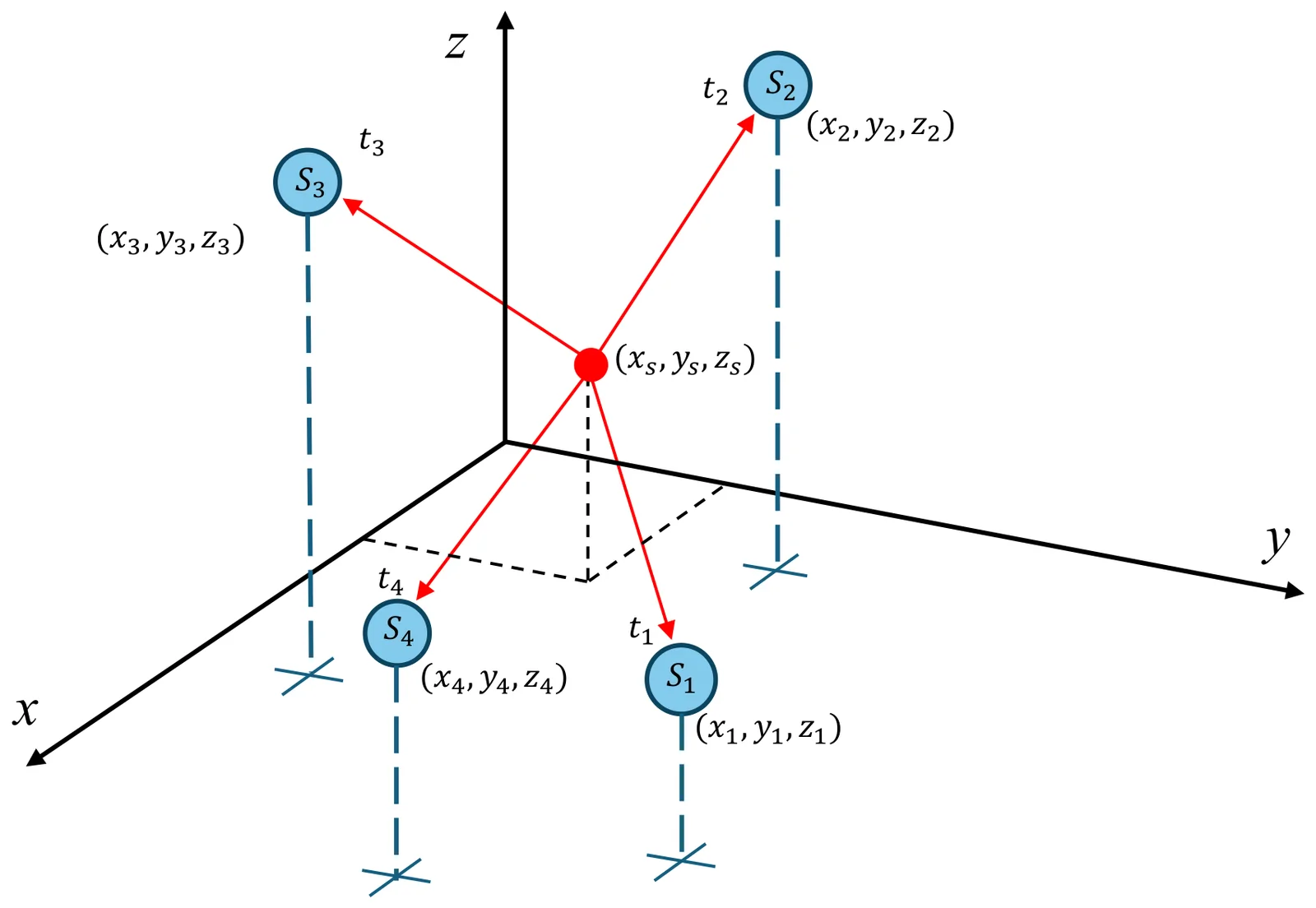
The time difference of arrival (TDOA) localization setup. The unknown spatial coordinates of the source, denoted by $p_{S}=\left(x_{S},y_{S},z_{S}\right)$, are to be estimated. Each receiver node $S_{i}$ is deployed at a known position $p_{i}=\left(x_{i},y_{i},z_{i}\right)$. The source emits an acoustic or electromagnetic signal at an unknown emission time $t_{S}$, which is subsequently detected by sensor $S_{i}$ at the arrival instant $t_{i}$.

The TDOA-based target localization setup is illustrated in Figure 1. Let $\left(x_{S},y_{S},z_{S}\right)$ denote the unknown source location, and let $\left(x_{i},y_{i},z_{i}\right)$ represent the known positions of the sensors $S_{i}$ for i=1,2,…,N (where N≥3). The source emits a signal at an unknown instant $t_{S}$, which arrives at sensor $S_{i}$ at time $t_{i}$. The range $R_{i}$ between the source and the *i*th sensor is defined as

(1) $$R_{i}=\sqrt{{\left(x_{i}-x_{S}\right)}^{2}+{\left(y_{i}-y_{S}\right)}^{2}+{\left(z_{i}-z_{S}\right)}^{2}}.$$

This range can also be expressed as

(2) $$R_{i}=c\left(t_{i}-t_{S}\right),$$

where *c* denotes the signal propagation speed in the medium (e.g., the speed of sound or light). Consequently, the range differences $d_{ij}$ can be directly computed from the arrival time measurements $t_{i}$ and $t_{j}$ via

(3) $$d_{ij}=R_{i}-R_{j}=c\left(t_{i}-t_{j}\right).$$

Given the measured range differences $d_{ij}$ and the known sensor coordinates $\left(x_{i},y_{i},z_{i}\right)$, the objective is to estimate the unknown source location.

### 2.2. Preliminary Estimation Process

Conventional (E-)COM-W methods operate within a two-dimensional search space under the assumption that the source lies on a known horizontal plane, specifically constraining the target coordinates to $p_{S}=\left(x_{S},y_{S},0\right)$ with $z_{S}=0$. To perform localization in this plane, a subset comprising three sensors, $S_{1}$, $S_{2}$, and $S_{3}$, is utilized. Following algebraic manipulation of (1) and (3), a quadratic equation is obtained for the range $R_{1}$:

(4) $$aR_{1}^{2}+bR_{1}+c=0,$$

where the coefficients *a*, *b*, and *c* can be computed from the sensor positions and the measured arrival times. Solving the quadratic equation in (4) yields the range $R_{1}$, from which the target position estimate $p_{S}$ is subsequently determined. For the complete derivation, please refer to [33].

Notice that, depending on the discriminant of (4), three distinct cases are to be considered:

- A single unique root: The single real solution $R_{1}$ is used to calculate $p_{S}$.
- No real roots: Consistent with the conventional COM-W method, the approximation $R_{1}=-b/\left(2a\right)$ is utilized to determine $p_{S}$.
- Two distinct real roots: Let the two roots of the quadratic equation be denoted as $R_{1}^{\left(1\right)}$ and $R_{1}^{\left(2\right)}$. The conventional COM-W method employs a heuristic root selection strategy that always selects the larger root to compute the target position $p_{S}$. Consequently, this approach can produce erroneous results depending on the specific geometric layout of the sensors. To address this limitation, the E-COM-W method computes two distinct position estimates, $p_{S}^{\left(1\right)}$ and $p_{S}^{\left(2\right)}$, corresponding to the two candidate roots, along with their respective overall error values. Ultimately, E-COM-W selects the candidate position exhibiting the smaller overall error as the final estimate for $p_{S}$.

The overall error for a position estimate $\left({\hat{x}}_{S},{\hat{y}}_{S},{\hat{z}}_{S}\right)$ is evaluated using all available measurements, as opposed to being restricted to sensors $S_{1},S_{2}$, and $S_{3}$, as follows:

(5) $$e=\sum_{i=1}^{N}{\left(R_{i}-{\hat{R}}_{i}\right)}^{2},$$

where the theoretical range $R_{i}$ is calculated as

(6) $$R_{i}=\sqrt{{\left(x_{i}-{\hat{x}}_{S}\right)}^{2}+{\left(y_{i}-{\hat{y}}_{S}\right)}^{2}+{\left(z_{i}-{\hat{z}}_{S}\right)}^{2}},$$

while ${\hat{R}}_{i}$ is the estimated range between the source and the *i*th sensor, defined as

(7) $${\hat{R}}_{i}=c\left(t_{i}-t_{S}\right),$$

with the signal emission time $t_{S}$ expressed as

(8) $$t_{S}=t_{1}-\frac{R_{1}}{c}.$$

### 2.3. Combination of the Preliminary Estimates

If the number of sensors is *N*, then the total number of sensor triplets, used in the previous step to calculate the preliminary position estimates, is given by:

(9) M=N3.

(E-)COM-W computes all preliminary position estimates $p_{m}$, with the corresponding Cramér–Rao lower bound (CRLB) metric ${\mathrm{CRLB}}_{m}$, for each unique sensor triplet m∈{1,2,…,M}. The final source position estimate is then computed as a weighted average:

(10) $${\hat{p}}_{S}=\sum_{m=1}^{M}w_{m}p_{m},$$

where the weight $w_{m}$ is defined as

(11) $$w_{m}=\frac{\frac{1}{{\mathrm{CRLB}}_{m}}}{\sum_{n=1}^{M}\frac{1}{{\mathrm{CRLB}}_{n}}}.$$

Notice that ${\mathrm{CRLB}}_{m}$ is computed from the positions of the sensors in the triplet *m* and the estimated preliminary position $p_{m}$, using a formula proposed in [33].

### 2.4. Performance Degradation of (E-)COM-W

**Figure 2:**
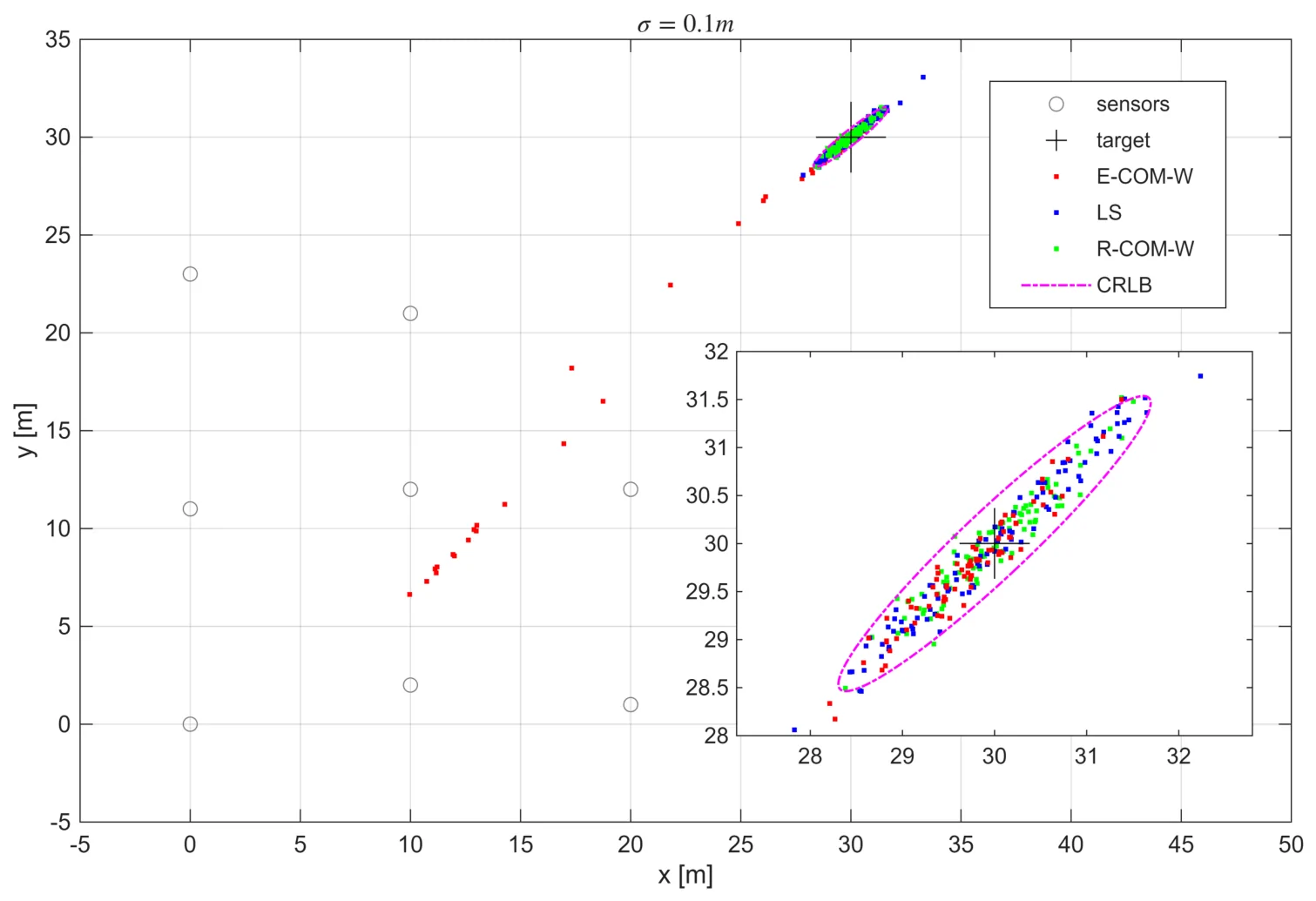
The localization case study with 300 experiments for σ=0.1m.

In this section, the noise-dependent performance limitations of the (E-)COM-W method are analyzed. The simulation topology for the case study is illustrated in Figure 2, which features eight sensor nodes denoted by gray circles and the true target position marked by black crosshairs. For presentation simplicity, the target and the sensors are coplanar, lying in the xy-plane (i.e., $z_{i}=0$ for i∈{1,2,…,8}). For each experiment, the ideal arrival times ${\bar{t}}_{i}$ are geometrically derived from the network topology, and zero-mean Gaussian noise $n_{i}∼N\left(0,\sigma_{t}^{2}\right)$ is added to generate the simulated measurements $t_{i}$. In the following discussions and figures, measurement discrepancies are characterized using the more intuitive meter-scale equivalent standard deviation, defined as $\sigma =c\cdot \sigma_{t}$.

From these noisy measurements, the target coordinates are estimated using E-COM-W, a conventional iterative least squares (LS) method, as well as the proposed R-COM-W approach. For each noise level, 300 independent trials were conducted. Figure 2 depicts the resulting position estimates for σ=0.1m alongside the 99% confidence error ellipse derived analytically from the Cramér–Rao lower bound (CRLB). It is apparent that while the majority of the estimates produced by the LS and the proposed R-COM-W methods are clustered tightly within the theoretical error ellipse, several estimates from the E-COM-W method exhibit significantly higher localization errors.

**Figure 3:**
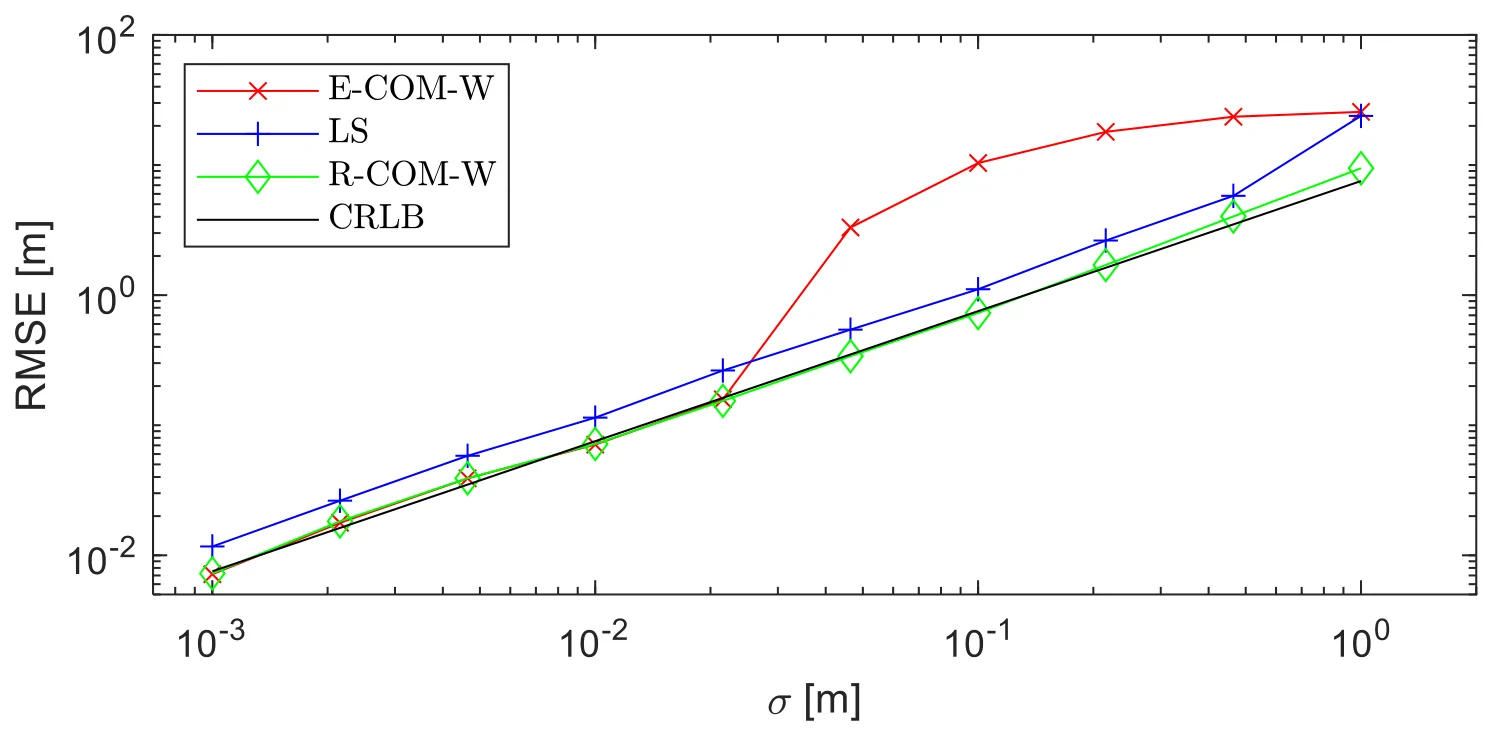
The RMSE as a function of measurement noise.

The noise-dependent performance of the evaluated methods is shown in Figure 3, where the noise level σ is varied from 1mm to 1m. The root mean square error (RMSE) calculated for each noise level is plotted alongside the theoretical minimum RMSE derived from the Cramér–Rao lower bound (CRLB).

The results demonstrate that the conventional (E-)COM-W method achieves excellent performance at low noise levels, closely approaching the CRLB when σ<0.04m. However, once the noise exceeds this threshold, its estimation error increases sharply, highlighting its vulnerability to higher-noise regimes. Although the iterative least squares (LS) algorithm maintains a consistent, steady trend, its error remains visibly above the CRLB across all noise conditions. In contrast, the performance of the proposed R-COM-W method remains unimpaired by increasing noise; its RMSE closely tracks the theoretical CRLB throughout the entire evaluated range.

Crucially, the noise threshold (σ≈0.04m) at which the suboptimal behavior of (E-)COM-W begins to emerge aligns closely with the typical measurement noise levels of practical acoustic and ultra-wideband (UWB) ranging technologies, which substantiates the practical significance of the problem.

**Figure 4:**
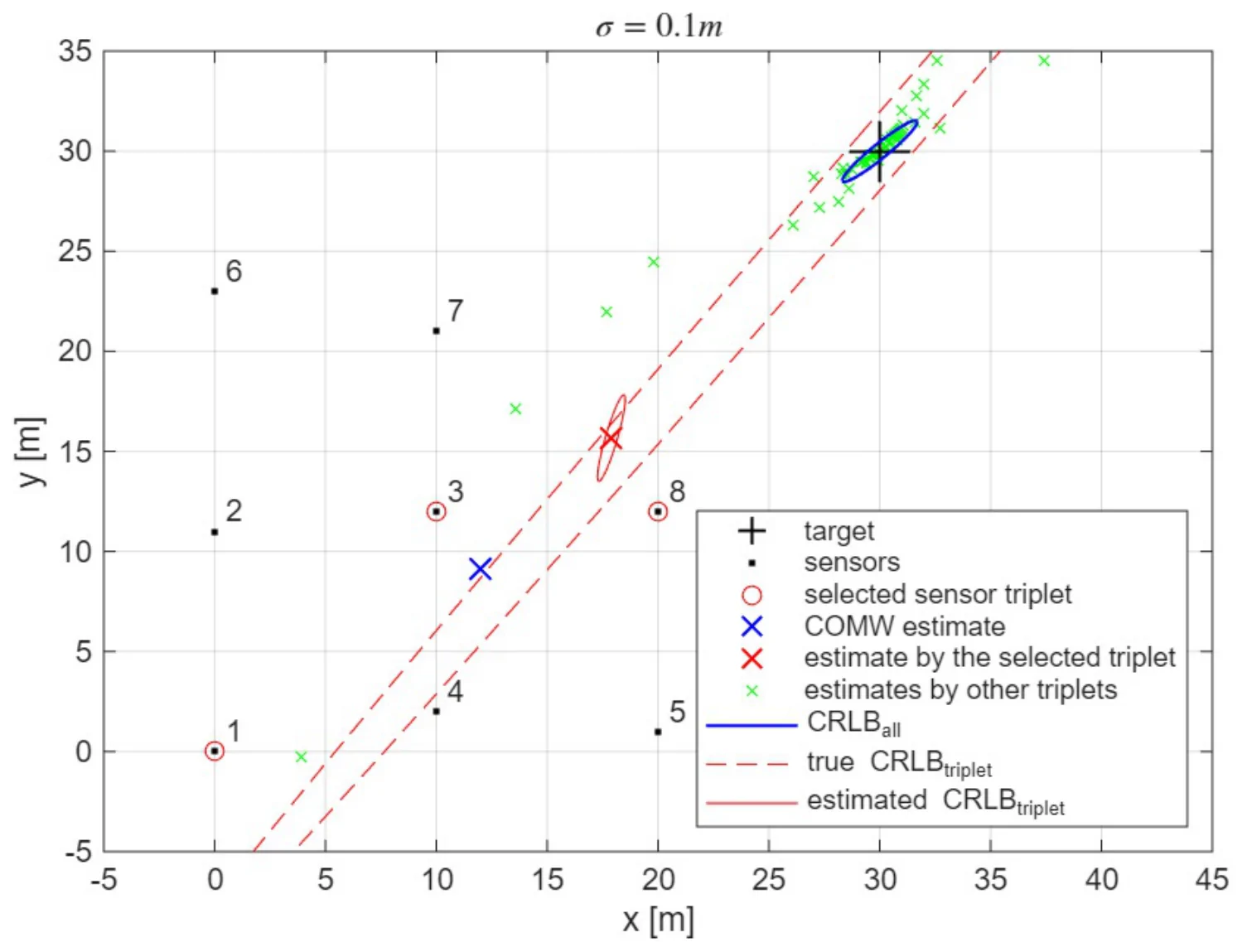
Details of the (E-)COM-W estimation process for σ=0.1m.

The underlying mechanism causing the suboptimal performance of (E-)COM-W is illustrated in Figure 4, which provides a detailed breakdown of a single position estimation instance. In this representative trial, the (E-)COM-W method exhibits markedly inferior performance, yielding a final coordinate estimate of ${\hat{p}}_{S}=\left(12.0\;m,9.2\;m\right)$ (marked by the blue ‘x’). This estimate lies a considerable distance from the blue ellipse, which represents the 99% confidence error boundary analytically derived from the Cramér–Rao lower bound (CRLB) at the true target position using the complete sensor configuration.

In the estimation process, the (E-)COM-W method evaluates all possible combinations of sensor triplets to compute the preliminary position estimates. In this specific scenario, M=56 unique triplets were processed, with their corresponding preliminary estimates denoted by the green crosses in Figure 4.

Consider the sensor triplet (1,3,8), highlighted by the red circles in Figure 4. This specific subset exhibits severe geometric dilution of precision (GDOP), as evidenced by the expansive error ellipse drawn with a dashed red line. Its theoretical Cramér–Rao lower bound (CRLB) evaluates to $326\;m^{2}$, implying that a minor measurement variance of $\sigma^{2}=0.01\;m^{2}$ amplifies to a position estimation variance of $326\;m^{2}$. The preliminary estimate derived from this geometric layout is marked by a red ‘x’ at coordinates ${\hat{p}}_{m}=\left(17.9\;m,15.7\;m\right)$. Due to its obvious inaccuracy, this estimate should ideally be assigned a negligible fusion weight $w_{m}$.

However, during the weight aggregation phase, the fusion weight $w_{m}$ for each preliminary estimate ${\hat{p}}_{m}$ is computed using its *estimated* CRLB (${\hat{\mathrm{CRLB}}}_{m}$) in (11). Crucially, while the true CRLB must be evaluated at the actual target position, this location is inherently unknown; hence, the bound is approximated at the estimated coordinates ${\hat{p}}_{m}$. In this example, the estimated ${\hat{\mathrm{CRLB}}}_{m}$ evaluated at ${\hat{p}}_{m}$ is only $0.6\;m^{2}$, as visualized by the solid red error ellipse. This estimated variance ($0.6\;m^{2}$) is substantially lower than the true underlying CRLB ($326\;m^{2}$), erroneously inflating its assigned weight by a factor of more than 500.

In this illustrative case study, the triplet exhibiting the most extreme performance degradation was (1,3,4), which produced a preliminary position estimate of ${\hat{p}}_{m}=\left(4.9\;m,-0.2\;m\right)$. The corresponding erroneously estimated weight exceeded its ideal theoretical value by a factor of more than 10,000 (this specific instance is omitted from Figure 4 to maintain visual clarity).

More generally, sensor subsets characterized by a high true CRLB are inherently prone to generating poor preliminary position estimates. As the measurement noise escalates, these preliminary estimates deviate further from the true source location, which subsequently degrades the accuracy of the locally evaluated CRLB approximations. In the critical scenario where the estimated ${\hat{\mathrm{CRLB}}}_{m}$ is severely underestimated relative to the true underlying CRLB, a highly inaccurate position estimate is assigned an artificially inflated weight $w_{m}$. Consequently, this poorly resolved estimate exerts a dominant, detrimental impact on the final aggregated position result.

## 3. Robust Combined Weighted Method for TDOA-Based Localization

This section introduces the proposed robust R-COM-W method. First, the preliminary position estimation process is detailed for the three-dimensional (3D) case. Subsequently, the combination framework designed to robustly aggregate these estimates is presented.

### 3.1. Preliminary Estimation Process in Three Dimensions

While the conventional (E-)COM-W methods are restricted to a two-dimensional search space, as detailed in Section 2.2, this paper extends the localization framework to three dimensions, allowing the full estimation of the spatial coordinate vector $p_{S}=\left(x_{S},y_{S},z_{S}\right)$. This three-dimensional solution is derived by maintaining structural and notational consistency with the logical framework originally established for the two-dimensional case in [33].

In three dimensions, a minimum of four sensors, $S_{1},S_{2},S_{3}$, and $S_{4}$, are required. From (1) and (3), the following equations can be derived:

(12) $$\begin{array}{cc} {\left(x_{1}-x_{S}\right)}^{2}+{\left(y_{1}-y_{S}\right)}^{2}+{\left(z_{1}-z_{S}\right)}^{2} & =R_{1}^{2}, \end{array}$$

(13) $$\begin{array}{cc} {\left(x_{2}-x_{S}\right)}^{2}+{\left(y_{2}-y_{S}\right)}^{2}+{\left(z_{2}-z_{S}\right)}^{2} & ={\left(R_{1}+d_{21}\right)}^{2}, \end{array}$$

(14) $$\begin{array}{cc} {\left(x_{3}-x_{S}\right)}^{2}+{\left(y_{3}-y_{S}\right)}^{2}+{\left(z_{3}-z_{S}\right)}^{2} & ={\left(R_{1}+d_{31}\right)}^{2}, \end{array}$$

(15) $$\begin{array}{cc} {\left(x_{4}-x_{S}\right)}^{2}+{\left(y_{4}-y_{S}\right)}^{2}+{\left(z_{4}-z_{S}\right)}^{2} & ={\left(R_{1}+d_{41}\right)}^{2}. \end{array}$$

Subtracting (12) from Equations (13)–(15) and performing straightforward algebraic manipulations yields the following system of linear equations: 

(16) $$A\left[\begin{array}{c} x_{S}\\ y_{S}\\ z_{S} \end{array}\right]=\frac{1}{2}\left[\begin{array}{c} 2R_{1}d_{21}+Q_{2}\\ 2R_{1}d_{31}+Q_{3}\\ 2R_{1}d_{41}+Q_{4} \end{array}\right],$$

where 

(17) $$A=\left[\begin{array}{ccc} x_{1}-x_{2} & y_{1}-y_{2} & z_{1}-z_{2}\\ x_{1}-x_{3} & y_{1}-y_{3} & z_{1}-z_{3}\\ x_{1}-x_{4} & y_{1}-y_{4} & z_{1}-z_{4} \end{array}\right]$$

is a matrix calculated from the sensor positions, which is required to be invertible, and 

(18) $$Q_{i}=d_{i1}^{2}+x_{1}^{2}+y_{1}^{2}+z_{1}^{2}-\left(x_{i}^{2}+y_{i}^{2}+z_{i}^{2}\right)$$

is computed from the sensor positions and the measurements. By defining the vectors $Q={\left[Q_{2},Q_{3},Q_{4}\right]}^{T}$ and $d={\left[d_{21},d_{31},d_{41}\right]}^{T}$, the position estimate can be expressed as

(19) $$p_{S}=\left[\begin{array}{c} x_{S}\\ y_{S}\\ z_{S} \end{array}\right]=R_{1}A^{-1}d+\frac{1}{2}A^{-1}Q=R_{1}\left[\begin{array}{c} m\\ q\\ r \end{array}\right]+\left[\begin{array}{c} n\\ t\\ u \end{array}\right],$$

where the intermediate vectors

(20) $$\left[\begin{array}{c} m\\ q\\ r \end{array}\right]=A^{-1}d\;\mathrm{and}\;\left[\begin{array}{c} n\\ t\\ u \end{array}\right]=\frac{1}{2}A^{-1}Q$$

are computed from known sensor configurations and the measured range differences $d_{i1}$.

Note that (20) requires the inversion of matrix *A*. To avoid numerical instability when *A* is singular or near-singular, the condition number of *A* is monitored, and sensor quartets with high condition numbers (e.g., $\mathrm{cond}\left(A\right)>10^{6}$) are excluded from the estimation process.

Substituting $x_{S}$, $y_{S}$, and $z_{S}$, as calculated from (19), into (12) yields the quadratic equation

(21) $$aR_{1}^{2}+bR_{1}+c=0,$$

where

(22) $$\begin{array}{cc} a & =1-m^{2}-q^{2}-r^{2}, \end{array}$$

(23) $$\begin{array}{cc} b & =2\left(m\left(x_{1}-n\right)+q\left(y_{1}-t\right)+r\left(z_{1}-u\right)\right), \end{array}$$

(24) $$\begin{array}{cc} c & =-{\left(n-x_{1}\right)}^{2}-{\left(t-y_{1}\right)}^{2}-{\left(u-z_{1}\right)}^{2}. \end{array}$$

Solving the quadratic equation in (21) yields the range $R_{1}$, which is subsequently substituted into (19) to obtain the target position estimate $p_{S}$. Depending on the discriminant of (21), three distinct scenarios are evaluated:

- No real roots: The range is approximated using the parabola vertex $R_{1}=-b/\left(2a\right)$, which is then used to compute the position estimate.
- A single unique root: The unique real solution is directly applied to determine the position estimate.
- Two distinct real roots: Both real roots are evaluated, yielding two candidate position estimates.

For each computed position estimate $p_{S}^{\left(i\right)}$ the corresponding Cramér–Rao lower bound (CRLB) metric ${\hat{\mathrm{CRLB}}}_{i}$ and the consensus value $C_{i}$ are evaluated and stored alongside the candidate coordinates. While the CRLB quantifies the theoretical lower bound on the variance of the estimate, the consensus value $C^{\left(i\right)}$ assesses the quality of the estimate relative to the actual measurements. The consensus value is introduced in detail in Section 3.2.

### 3.2. The Consensus Value

The consensus value at a given position $p_{S}$ represents the number of measurements $t_{i}$ that collectively support the hypothesis that $p_{S}$ is the true target location, given a maximum measurement error of Δt, or, equivalently, a maximum range error of Δs=cΔt [11]. Given a candidate target position $p_{S}$, the ranges $R_{i}$ can be computed using (1). From each arrival time measurement $t_{i}$, the corresponding emission time $T_{i}$ can be estimated as

(25) $$T_{i}=t_{i}-\frac{R_{i}}{c}.$$

Under ideal conditions, where $p_{S}$ matches the true target coordinates and the measurement noise is zero, all emission time estimates converge such that $T_{i}=t_{S}$. If the measurement noise is bounded by Δt, then $|T_{i}-t_{S}|\leq \Delta t$. Consequently, the correct emission time estimates $T_{i}$ form a cluster along the timeline centered at $t_{S}$, spanning a maximum width of w=2Δt.

**Figure 5:**
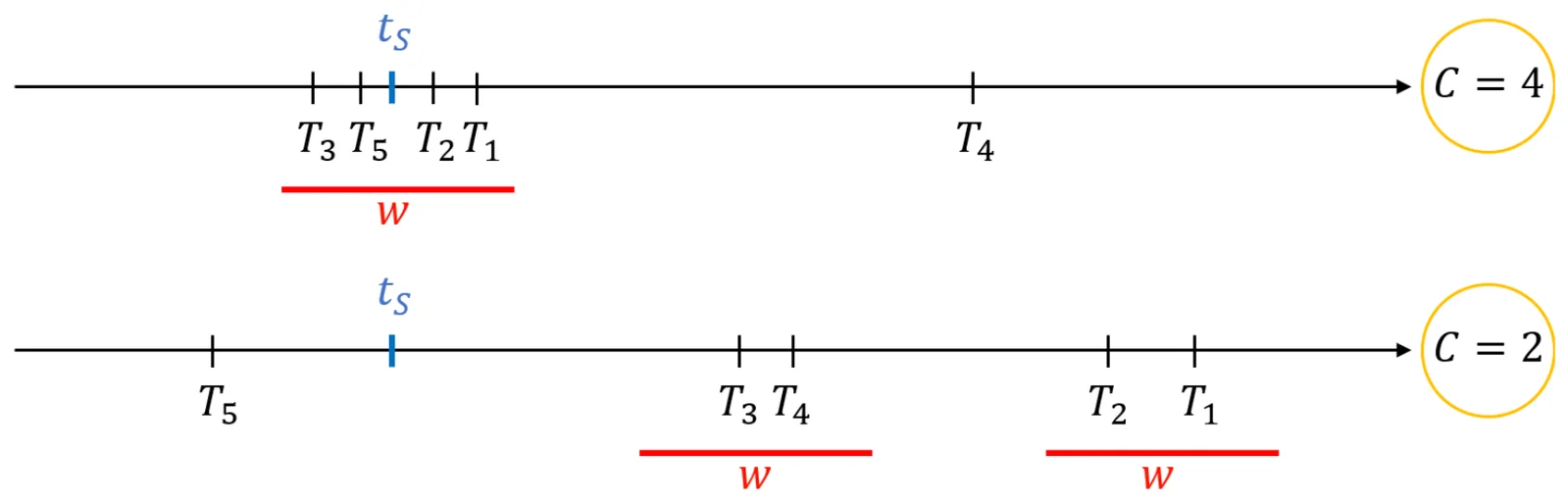
Illustration of the consensus metric computation using five measurements. Upper timeline: evaluated at the true target position, four consistent (but noisy) measurements support the location hypothesis, while measurement 4 is an outlier, yielding a consensus value of C=4. Lower timeline: evaluated at an incorrect candidate position, the emission time estimates $T_{i}$ scatter sparsely across the timeline, forming only small, disjoint groups with a maximum consensus value of C=2.

If a subset of *k* measurements is corrupted by large outlier errors, their corresponding $T_{i}$ estimates will deviate significantly from this cluster; however, the valid cluster will retain N−k members. This scenario is illustrated in the upper timeline of Figure 5, which depicts one outlier alongside four consistent measurements. Conversely, if $p_{S}$ deviates from the true target position, it is highly improbable for a large number of $T_{i}$ estimates to form a dense cluster. Instead, they will scatter sparsely along the timeline, leaving only small, disjoint groups, as shown in the lower timeline of Figure 5.

An efficient method for computing the consensus value is outlined in Algorithm 1. The algorithm processes a sorted vector of time measurements using left and right indices (*L* and *R*) to identify the boundaries of the largest subset of consistent measurements. The maximum window cardinality R−L+1 encountered during the traversal is recorded as the consensus value *C*. Sorting requires O(NlogN) operations in the worst case, whereas the index search iteration exhibits O(N) time complexity, yielding an overall worst-case computational complexity of O(NlogN).
**Algorithm 1:** Calculation of the Consensus Value**Data:** Input array of measurements $T_{i}$ of size *N*, consensus window width *w*
**Result:** Consensus value *C*
**sort** $T_{i}$ in increasing order;
L←1;R←1;**while** R<N **do**
**increment** *R* **while** $T_{R}\leq T_{L}+w$ **and** R=N; C←R−L+1; L←L+1;
**end**


Notice that the width parameter *w* is directly derived from the ranging accuracy. In practice, the error budget of the sensors is typically known or can be reliably estimated. If the variance of the time measurements $\sigma_{t}^{2}$ is known, then the bound Δt can be estimated as $3\sigma_{t}$, yielding a width parameter of $w=6\sigma_{t}$. Alternatively, in many practical scenarios, the maximum measurement error Δt can be empirically estimated [11], and w=2Δt can be utilized. Fortunately, the consensus value in real-world applications is not highly sensitive to the precise selection of *w*, meaning a rough approximation of Δt is entirely sufficient [35], as will be illustrated in Section 4.

The window width of the consensus function is defined in the time domain. Because distance serves as a more intuitive metric, in the presentation of the experiments we employ the distance-equivalent window width, computed as $w_{d}=c\cdot w$, where *c* is the speed of sound.

### 3.3. Robust Combination of the Preliminary Estimates

In three dimensions, sensor quartets are used to calculate the preliminary estimates, as described in Section 3.1. If the number of sensors is *N*, then the total number of sensor quartets is

(26) M=N4.

During the preliminary calculation phase, the R-COM-W method computes either one or two position estimates for each sensor combination m∈{1,2,…,M} (yielding two candidate estimates for cases with two real roots and a single estimate otherwise). Thus, the input to the subsequent combination phase is a set of 3-tuples $\left(p_{S}^{\left(i\right)},\hat{{\mathrm{CRLB}}_{i}},C_{i}\right)$, where $i\in \{1,2,\ldots ,M^{\prime }\}$ with $M^{\prime }\geq M$).

The maximum consensus metric among all candidate configurations is defined as $C_{\max}=\max_{i}C_{i}$. Let the consensus set CS encompass the indices *k* that satisfy $C_{k}=C_{\max}$. The final target position estimate is then evaluated as the weighted average:

(27) $$p_{S}=\sum_{m\in \mathrm{CS}}w_{m}p_{S}^{\left(m\right)},$$

where $w_{m}$ denotes the normalized fusion weight derived from the estimated CRLB of the consistent sensor triplets (in the 2D case) or quartets (in the 3D case):

(28) $$w_{m}=\frac{1/{\hat{\mathrm{CRLB}}}_{m}}{\sum_{n\in \mathrm{CS}}1/{\hat{\mathrm{CRLB}}}_{n}},$$

and ${\hat{\mathrm{CRLB}}}_{m}$ is computed using the analytical formulations derived in [36], as follows:

Let $p_{j}={\left[x_{j},y_{j},z_{j}\right]}^{⊤},j=1,2,3,4$ denote the actual positions of the sensor quartet *m*, and let $p_{S}^{\left(m\right)}$ be the corresponding preliminary estimate. Define

(29) $$g_{j}=\frac{p_{S}^{\left(m\right)}-p_{j}}{∥p_{S}^{\left(m\right)}-p_{j}∥},j=1,2,3,4$$

and

(30) $$G=\left[\begin{array}{c} g_{2}^{T}-g_{1}^{⊤}\\ g_{3}^{T}-g_{1}^{⊤}\\ g_{4}^{T}-g_{1}^{⊤} \end{array}\right].$$

The Fisher information matrix is

(31) $$F=\frac{1}{c^{2}}G^{T}Q^{-1}G,$$

where *Q* is the noise covariance matrix

(32) $$Q=\left[\begin{array}{ccc} \sigma_{1}^{2}+\sigma_{2}^{2} & \sigma_{1}^{2} & \sigma_{1}^{2}\\ \sigma_{1}^{2} & \sigma_{1}^{2}+\sigma_{3}^{2} & \sigma_{1}^{2}\\ \sigma_{1}^{2} & \sigma_{1}^{2} & \sigma_{1}^{2}+\sigma_{4}^{2} \end{array}\right],$$

while $\sigma_{i}^{2}$ is the variance of measurement $t_{i}$. The target–receiver geometry is captured in G, while the measurement noise characteristics are modeled by Q. Specifically, the noise variance $\sigma_{i}^{2}$ depends on the source–sensor distance $R_{i}$ and is often described by the standard power-law path-loss model:

(33) $$\sigma_{i}^{2}=\sigma_{0}^{2}R_{i}^{\alpha },$$

where $\sigma_{0}^{2}$ is the baseline variance at unit distance, while α is the path-loss exponent (typically 2≤α≤4). Because the proposed algorithm relies exclusively on relative CRLB ratios, the global scale parameter $\sigma_{0}^{2}$ cancels out and can be set to unity ($\sigma_{0}^{2}=1$) without loss of generality. In practical deployments, environmental and hardware non-idealities—such as multipath propagation, clock jitter, and clock drift—frequently dominate distance-dependent path loss in contributing to measurement error variance. Consequently, isolating distance dependencies alone is often impractical. For this reason, we adopt the standard simplified model assuming equal noise variance across sensors, i.e., $\sigma_{i}=\sigma$, which provides a robust baseline without making overly restrictive channel assumptions. With this model, (21) is simplified to

(34) $$Q=\sigma^{2}\left[\begin{array}{ccc} 2 & 1 & 1\\ 1 & 2 & 1\\ 1 & 1 & 2 \end{array}\right].$$

where the exact value of the noise variance $\sigma^{2}$ does not affect the solution and can be set arbitrarily (e.g., $\sigma^{2}=1$).

The CRLB estimate is calculated as

(35) $${\hat{\mathrm{CRLB}}}_{m}=trace\left(F^{-1}\right).$$

As the number of deployed sensors *N* increases, the total number of evaluation combinations *M* escalates in proportion to $O(N^{D+1})$, where *D* represents the spatial dimension. For each combination, the consensus value is computed with a worst-case complexity of O(NlogN). Consequently, the overall worst-case computational complexity of the algorithm is $O(N^{D+2}\log N)$. This combinatorial expansion can severely impact computational latency. To mitigate this overhead in sensor-dense environments, the number of evaluated subsets can be constrained to a maximum threshold $M_{\max}$, where $M_{\max}$ sensor quartets are chosen at random. Under this bounded evaluation scheme, the total computational complexity reduces to $O(M_{\max}\cdot N\log N)$, or simply O(NlogN). In practice, setting $M_{\max}$ on the order of a few hundred provides a robust balance between localization accuracy and processing speed, as demonstrated in Section 4.

## 4. Evaluation

In this section, the performance of R-COM-W is evaluated using simulations and real data. The performance of R-COM-W is compared to that of COM-W, E-COM-W, and the LS methods. All evaluated methods are tested in 3D; thus, the original COM-W and E-COM-W algorithms were also extended to 3D by applying selected results from Section 2 as follows: preliminary estimates are computed in 3D using the formulation proposed in Section 3.1, while the weights are calculated via the CRLB formula described in Section 3.3.

### 4.1. Experimental Setup

**Figure 6:**
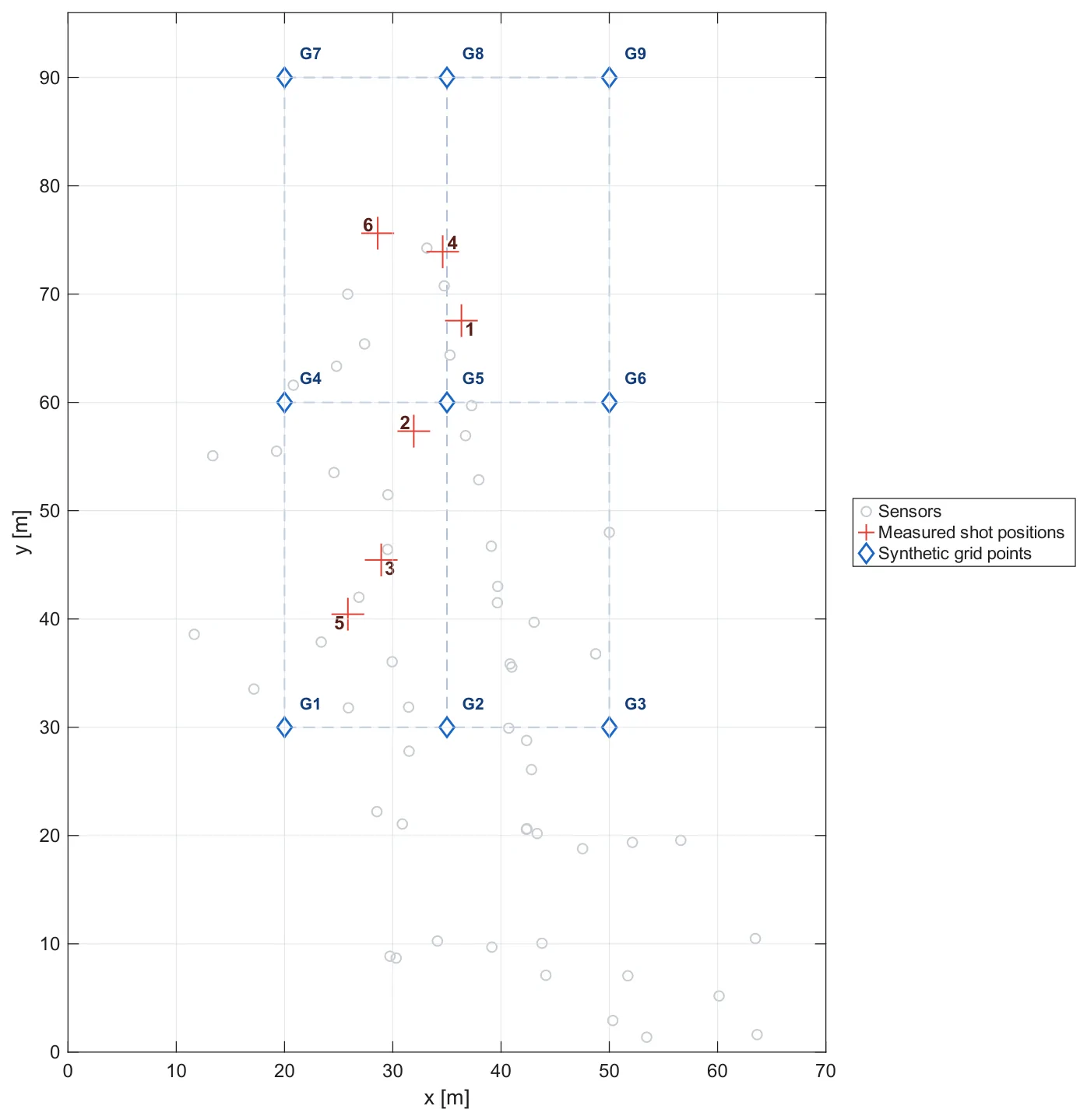
Measurement geometry in the horizontal plane, showing the 57 sensor positions, the six measured shot locations, and the 3×3 synthetic source grid used in the simulations. All synthetic sources are located at z=0m.

The experimental evaluation environment is illustrated in Figure 6. The sensor topology replicates the physical layout employed in the acoustic shooter localization experiment reported in [37], which comprises 57 sensor nodes deployed across a 70m×90m area. In addition to the sensor geometry, Figure 6 depicts nine test positions utilized in the simulation studies, as well as six actual shooter locations employed during the physical measurements.

To evaluate the performance of the proposed R-COM-W method, its localization accuracy and computational complexity are benchmarked against the least squares (LS) algorithm [38], as well as the conventional COM-W and E-COM-W methods. The actual implementation of the LS algorithm is a custom MATLAB implementation of a multi-start Levenberg–Marquardt nonlinear least squares estimator. To prevent potential non-convergence of the LS algorithm, initial search points were selected within a 5m radius of the true source locations, giving the LS benchmark a slight advantage during comparative evaluation.The MATLAB implementations of the COM-W, E-COM-W, and R-COM-W algorithms directly correspond to the formulations presented in Section 2 and Section 3.

All numerical experiments and simulation runs were executed using MATLAB R2025a on an HP EliteBook 660 G11 computer, equipped with an Intel Core Ultra 7 155U processor and 16GB of RAM.

### 4.2. Simulations

The synthetic robustness evaluation utilizes the 57-sensor network geometry illustrated in Figure 6. The source grid comprises nine distinct positions defined by the coordinates x∈{20,35,50}m, y∈{30,60,90}m, and a constant elevation of z=0m. Throughout these simulations, error-free arrival times were first computed based on the true sensor-source geometry. Subsequently, zero-mean additive Gaussian noise was introduced with distance-equivalent standard deviation of σ=0.2m. In each simulation trial, a subset of $N_{\mathrm{out}}$ sensors were randomly chosen as outliers, where $N_{\mathrm{out}}\in \left\{0,5,10,\ldots ,40\right\}$. For these selected sensors, an additional exponential noise term Exp(λ)—derived from the Saleh–Valenzuela channel model to reflect typical NLOS situations [39,40]—was added to the computed arrival times, with a mean distance-equivalent value of μ=1/λ=2m. The consistency window for the R-COM-W algorithm was $w_{d}=0.60$ m, and the maximum number of subsets evaluated was $M_{\max}=300$.

**Figure 7:**
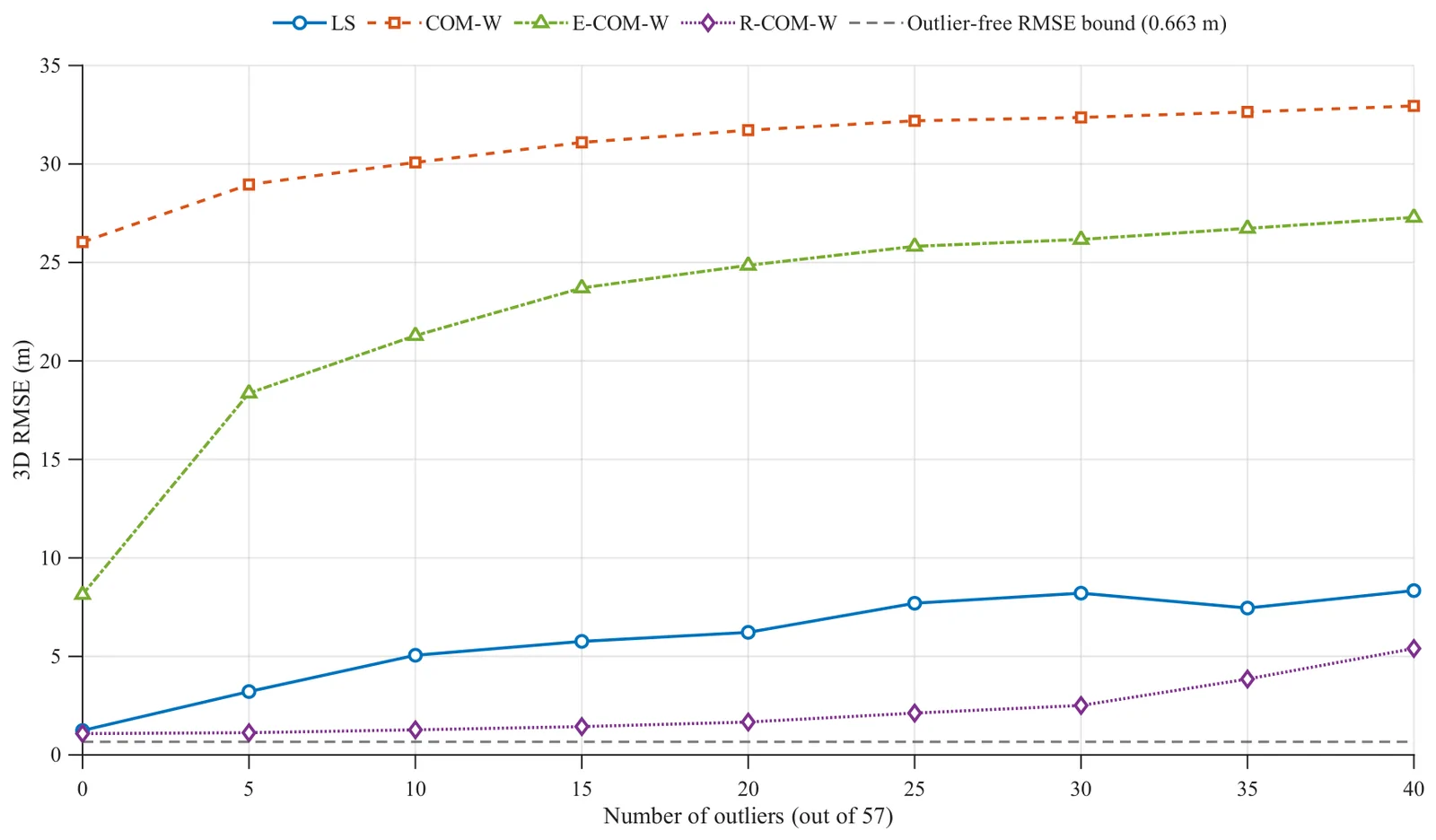
Root mean square localization error as a function of the outlier cardinality $N_{\mathrm{out}}$. The total number of sensors was N=57.

For each of the nine source positions and each outlier cardinality $N_{\mathrm{out}}$, a total of 100 independent Monte Carlo trials were generated (yielding 9×100=900 simulation runs for each value of $N_{\mathrm{out}}$), and the corresponding localization error was computed for each algorithm. The resulting root mean square errors (RMSEs) are summarized in Table 1 and plotted in Figure 7.

The results indicate that in the absence of outliers ($N_{\mathrm{out}}=0$), LS and R-COM-W perform well, yielding RMSEs of 1.2 m and 1.1 m, respectively. The overall theoretical RMSE bound for the outlier-free scenario is calculated as the root mean of the CRLB values across nine test points, with a result of 0.66 m. This demonstrates that the estimation performance of R-COM-W indeed closely approaches the theoretical minimum. COM-W and E-COM-W perform poorly even in this outlier-free scenario. When outlier measurements are introduced, the performance of the algorithms—except for R-COM-W—degrades rapidly. In contrast, R-COM-W exhibits robust performance, maintaining a localization error around 2.5 m (approximately 2.5 times that of the outlier-free case) even when the outlier cardinality $N_{\mathrm{out}}$ reaches 30, corresponding to over 50% contaminated measurements.

**Figure 8:**
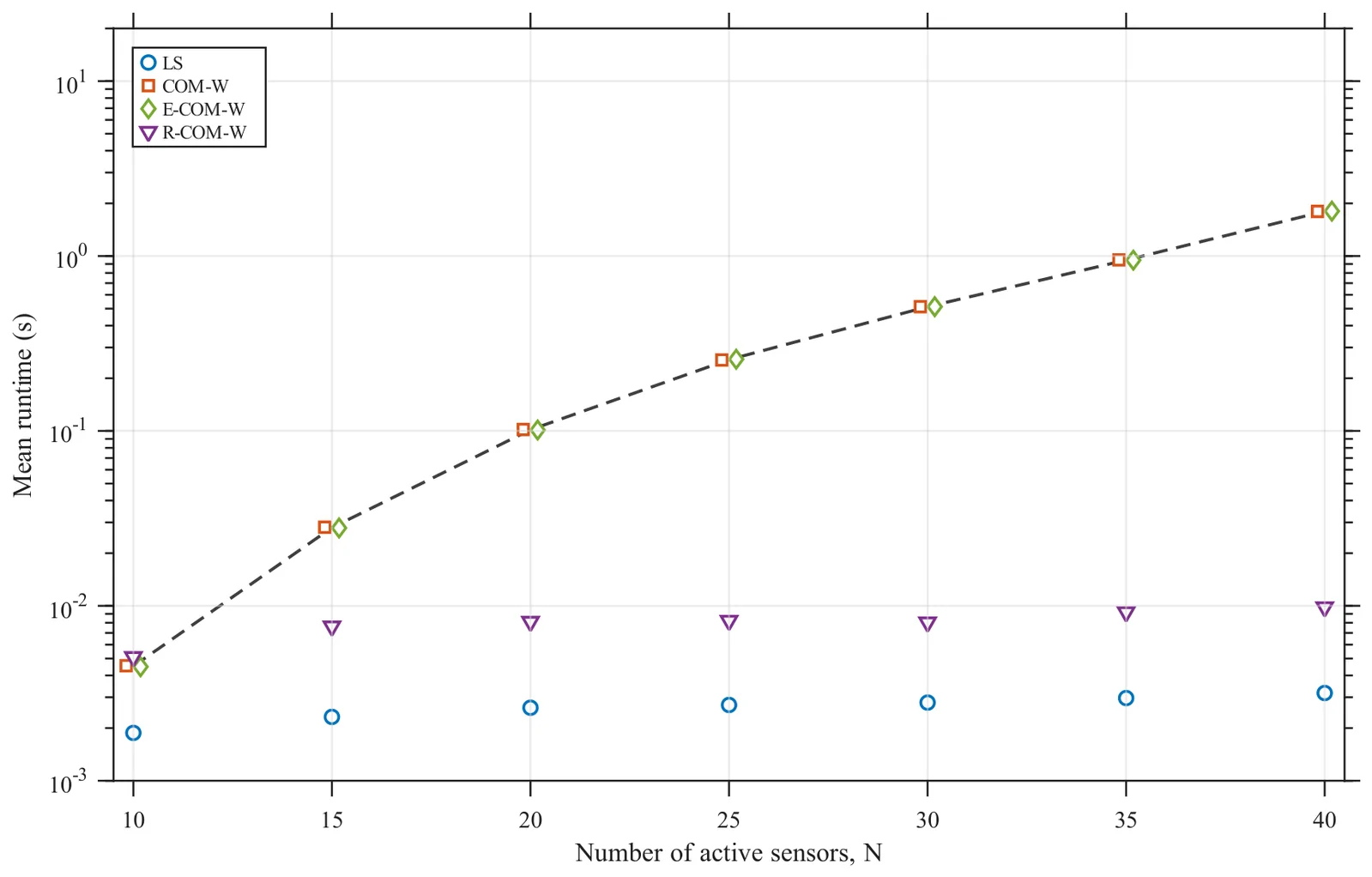
Runtime scales with active sensor count *N*.

The execution times of the evaluated algorithms are summarized in Table 2 and plotted in Figure 8 as a function of the number of sensors *N*. The execution time of the LS method exhibits minimal dependence on *N*, remaining nearly constant at an average of approximately 3ms. In contrast, the COM-W, and E-COM-W algorithms display highly similar computational scaling, with runtimes that can be closely approximated by a polynomial curve of the form $C\cdot N^{4.3}$, where $C\;=2.6\cdot 10^{-7}$. However, when R-COM-W is used with a restricted maximum number of random sensor quartets ($M_{\max}=300$), its computational demand is drastically reduced, requiring a maximum execution time of only 10ms.

To evaluate the dependence of R-COM-W performance on the maximum number of sensor quartets, $M_{\max}$, simulation experiments were conducted using the simulation setup with nine test positions, shown in Figure 6. In the experiments, four distinct scenarios, combining two distance-equivalent measurement noise levels ($\sigma_{d}=0.2\;m$ and $\sigma_{d}=0.3\;m$) with two outlier counts ($N_{\mathrm{out}}=0$ and $N_{\mathrm{out}}=10$), were utilized. The window width parameter was $w_{d}=0.6\;m$. $M_{\max}$ was varied from 10 to 10,000, and for each parameter configuration, 100 independent Monte Carlo runs were executed to compute the mean error and average computational time.

**Figure 9:**
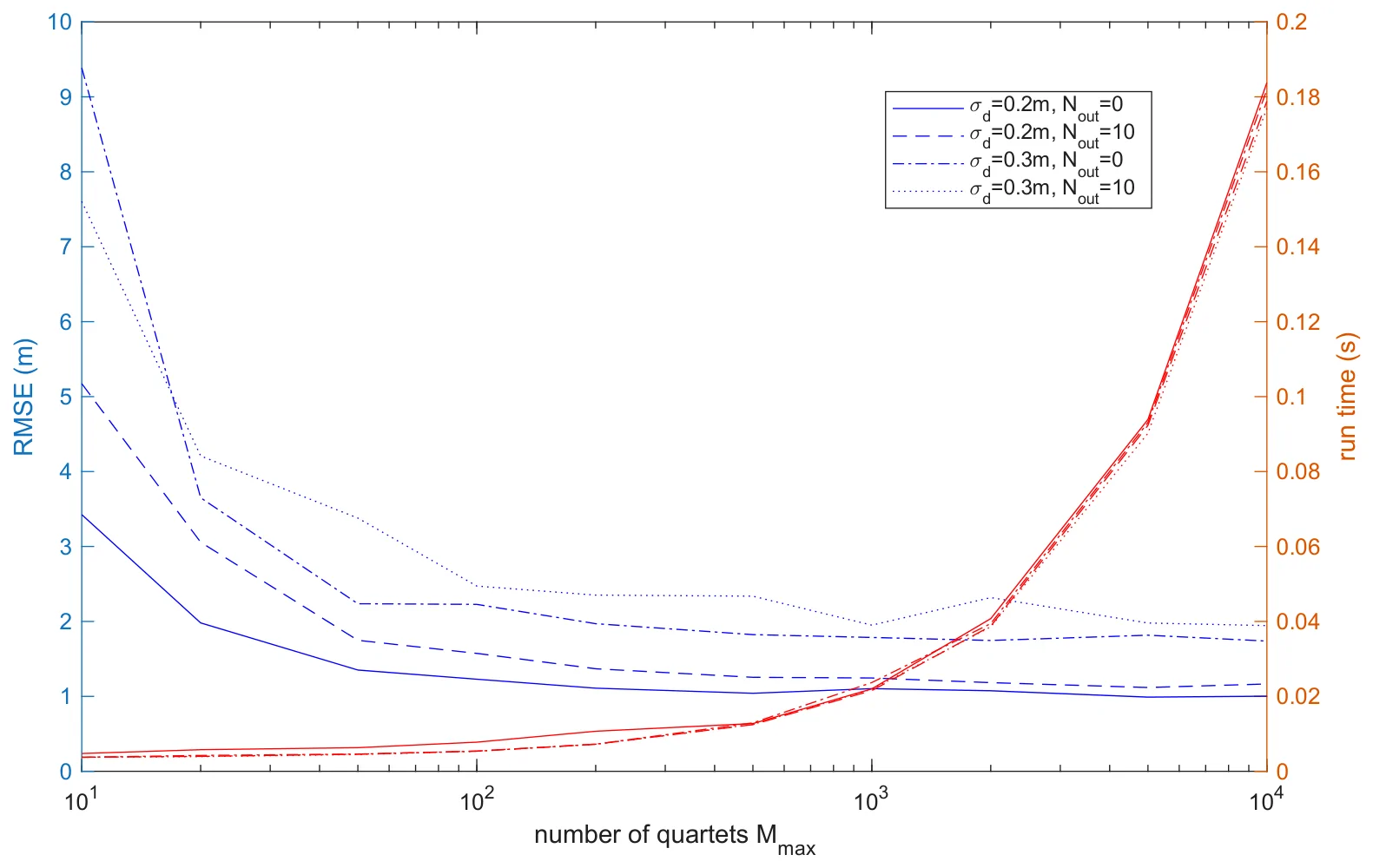
Estimation error and execution time as a function of the maximum number of sensor quartets, $M_{\max}$.

The results are plotted in Figure 9. As expected, the error decreases as the number of quartets increases. As shown in Figure 9, the error declines sharply for $M_{\max}<100$ and begins to plateau around $M_{\max}=200$. Further increasing $M_{\max}$ marginally improves estimation accuracy at the cost of additional execution time. Consequently, $M_{\max}=300$ serves as a suitable trade-off between speed and precision.

**Figure 10:**
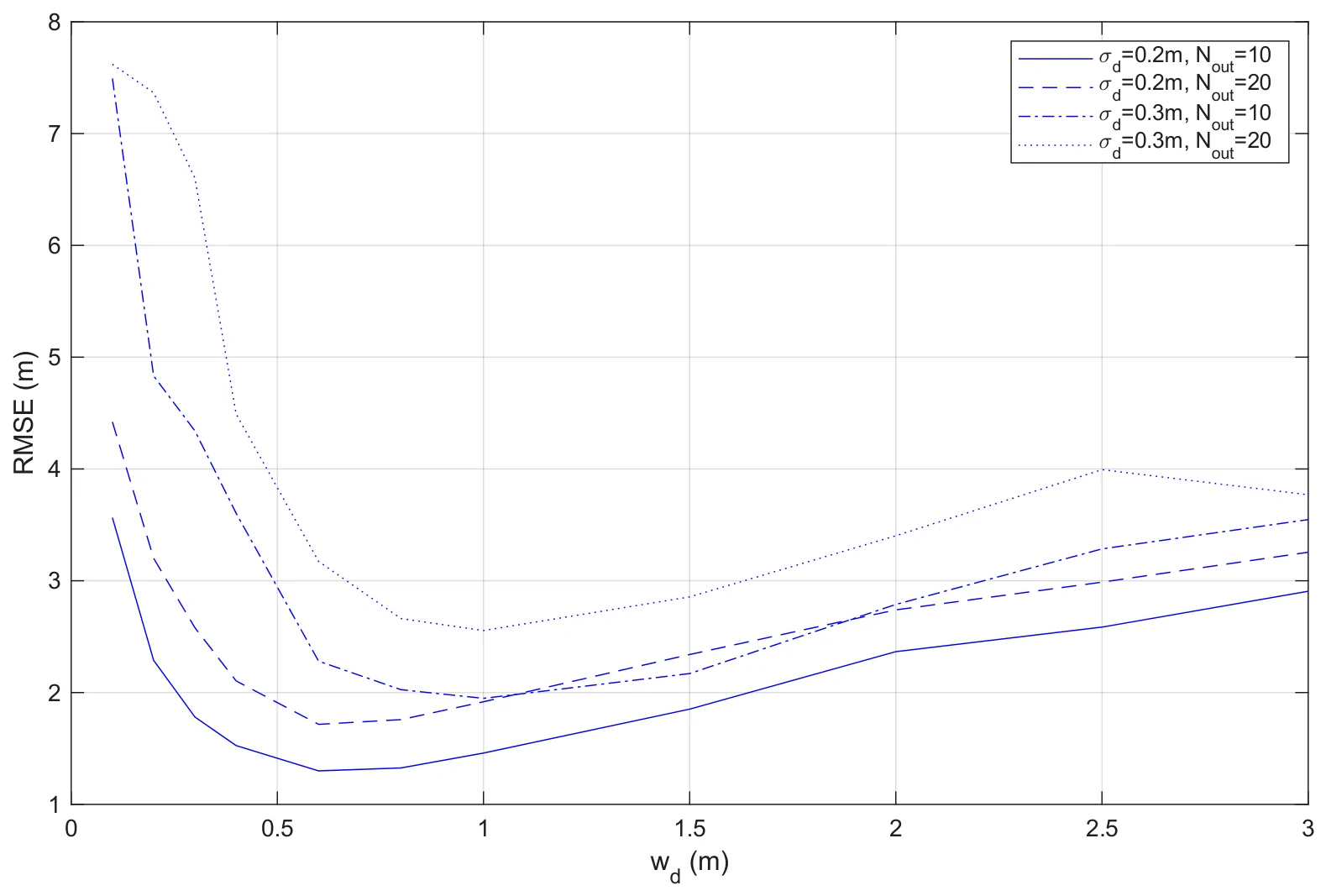
Estimation error as a function of distance-equivalent window width $w_{d}$.

To demonstrate that the proposed method is robust against the choice of window-width parameter $w_{d}$, an experiment was conducted using measurement noise levels $\sigma_{d}=0.2\;m$ and $\sigma_{d}=0.3\;m$ and outlier counts $N_{\mathrm{out}}=10$ and $N_{\mathrm{out}}=20$. Here, $M_{\max}$ was set to 300, while $w_{d}$ varied between 0.1m and 3.0m. When the window-width parameter $w_{d}$ is too small, even minor measurement noise can exclude valid measurements from the consensus window. This reduces the number of utilized sensors and degrades overall localization accuracy. Conversely, if $w_{d}$ is too large, outliers may be inadvertently included in the consensus sensor set, likewise impairing performance. As shown in Figure 10, the error curves exhibit a broad minimum from $w_{d}=2\sigma_{d}$ to approximately $w_{d}=6\sigma_{d}$, indicating low sensitivity to this parameter.

### 4.3. Measurements

**Figure 11:**
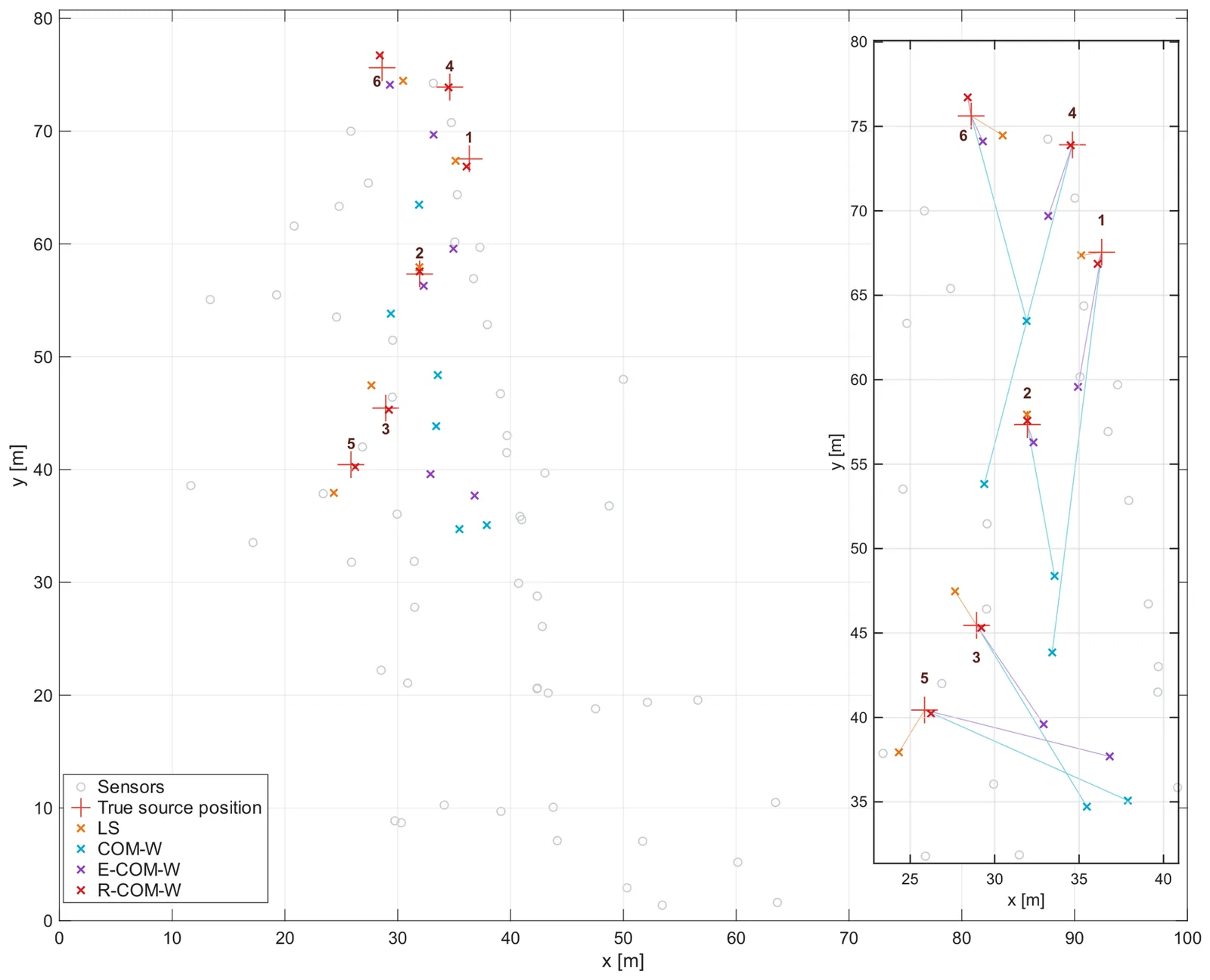
Geometric layout and localization results for the six measured shots. The big panel shows the full deployed sensor network, while the insert provides an enlarged view of the shooter region. Light gray open circles denote the deployed sensors, and red plus signs mark the surveyed shooter positions, numbered 1–6. Magenta, cyan, green, and blue crosses show the position estimates obtained with LS, COM-W, and E-COM-W, and R-COM-W, respectively. All four methods estimate the full three-dimensional source position; the figure displays the corresponding *x*–*y* projections.

Real measurements from the acoustic shooter localization experiment [37] were utilized to test R-COM-W. The sensor setup and the six test shooter positions are shown in Figure 11. The measurement location was a military training site simulating urban terrain, which resulted in severe echoes and a lack of line of sight (LOS) for many sensors. As a consequence, all six datasets contained significant numbers of outliers. Based on ground-truth shooter positions, offline analysis revealed that outliers comprised 26% to 67% of the total sensor readings (note that ground-truth data were excluded from the estimation process).

**Figure 12:**
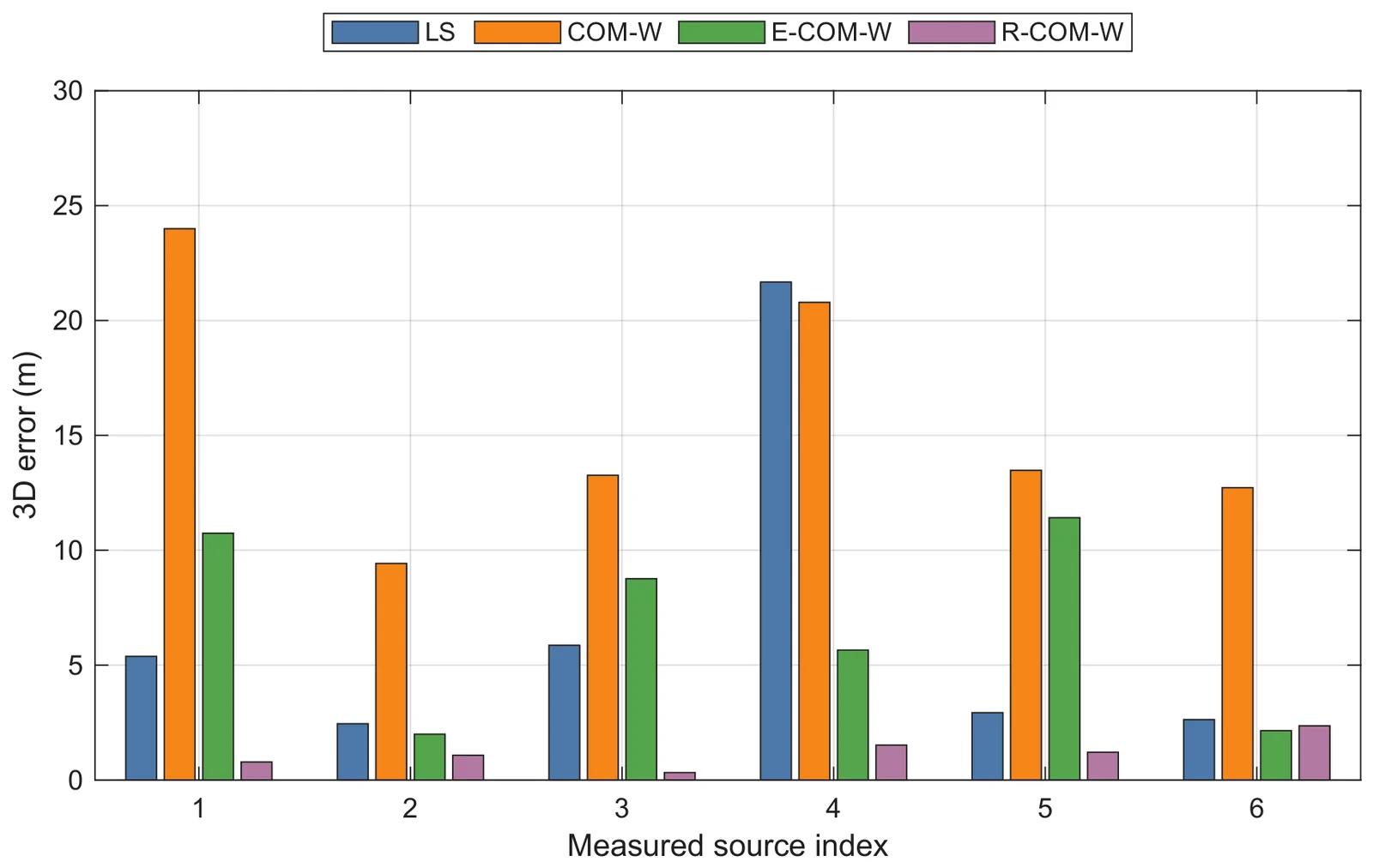
Estimation errors in the six-shot experiment.

The estimated upper bound for the equivalent measurement error of the sensors with line of sight was approximately 0.3 m; thus, the R-COM-W parameter was set to $w=2\times 0.3\;m/v_{\mathrm{sound}}=1.8\;\mathrm{ms}$. The maximum iteration number $M_{\max}$ was set to 300. The estimated positions for LS, COM-W, E-COM-W, and R-COM-W are shown in Figure 11, while the detailed results—including the 3D estimation errors and execution times—are presented in Table 3 and the estimation errors are also plotted in Figure 12.

As illustrated in Table 3, R-COM-W consistently achieved estimation errors of approximately 1 m—except for shot #6, where the error was 2.4 m—whereas the other evaluated methods generated significantly larger errors. LS and E-COM-W produced comparable outcomes, yielding a mean error of approximately 6.8 m, while COM-W exhibited a mean error exceeding 15 m. These results clearly demonstrate the superior performance of R-COM-W over conventional approaches.

LS was the fastest of the tested methods, with an average runtime of approximately 12 ms. The mean execution time required for R-COM-W to complete the estimation was approximately 19 ms (with $M_{\max}=300$), whereas COM-W and E-COM-W—without iteration restrictions applied—required more than 500 ms to run on average. Note, that without the restriction of $M_{\max}$, R-COM-W would require execution times within a similar range.

These results clearly demonstrate that while conventional methods yield substantial estimation errors in NLOS scenarios, R-COM-W exhibits robust performance in the presence of numerous outliers in real-world measurements, while maintaining a computational demand comparable to that of LS.

## 5. Conclusions

A robust version of COM-W (termed R-COM-W) is proposed. In the original (E-)COM-W methods, the first estimation phase utilizes sensor groups with the minimum required number of sensors (three for 2D and four for 3D) to provide preliminary estimates. In the second step, these preliminary estimates are combined to yield the final location estimate, using weights derived from the CRLB to circumvent the impact of potentially ill-conditioned sensor groups. However, as illustrated in this paper, the effectiveness of this approach diminishes as the measurement noise level rises.

To overcome this issue, we propose consensus numbers to identify and eliminate sensor groups providing inaccurate preliminary results based on their actual—rather than potential—performance. This same mechanism effectively mitigates outlier measurements as well.

The performance of the proposed R-COM-W method was evaluated against that of the constrained LS method as well as earlier variants of the COM-W family (COM-W and E-COM-W). Simulations modeling a challenging acoustic environment were conducted to assess estimation accuracy in the presence of outliers (e.g., caused by non-line-of-sight conditions) and to analyze execution speed. Furthermore, real-world acoustic shooter measurements obtained in an environment rich in non-line-of-sight propagation and reverberations were utilized to validate the proposed method.

Evaluated across both synthetic acoustic simulations and empirical real-world acoustic measurements, R-COM-W attains near-CRLB localization performance while exhibiting high robustness to measurement noise. Furthermore, R-COM-W demonstrates exceptional resilience against corrupted measurements—such as those induced by non-line-of-sight (NLOS) conditions—maintaining high estimation accuracy even when outliers constitute over 50% of the dataset.

A primary disadvantage of COM-W approaches is that their computational complexity scales as $O(N^{4.3})$ for 3D localization, which can severely impact latency in sensor-dense environments. To address this limitation, R-COM-W introduces an effective compromise by evaluating only a subset of all potential sensor groups. This approach dramatically reduces the computational workload while maintaining high accuracy, achieving execution speeds comparable to those of the least squares (LS) method.

## Figures and Tables

**Table 1:** Comparison of root mean square localization errors (RMSEs) in meters across different outlier cardinalities $N_{\mathrm{out}}$.

Number of Outliers ($N_{\mathrm{out}}$)	LS	COM-W	E-COM-W	R-COM-W
0	1.24	26.04	8.14	1.08
5	3.21	28.96	18.37	1.13
10	5.06	30.08	21.29	1.27
15	5.76	31.10	23.71	1.44
20	6.22	31.71	24.86	1.67
25	7.70	32.19	25.82	2.12
30	8.21	32.36	26.17	2.51
35	7.46	32.64	26.73	3.85
40	8.34	32.95	27.29	5.40

**Table 2:** Mean runtime of the tested algorithms as a function of the active sensor count *N*. The values are given in milliseconds.

*N*	LS	COM-W	E-COM-W	R-COM-W
10	1.9	4.5	4.5	5.1
15	2.3	28.2	27.9	7.6
20	2.6	101.9	101.2	8.1
25	2.7	254.4	257.1	8.2
30	2.8	513.1	513.4	8.0
35	3.0	950.1	946.7	9.2
40	3.2	1798.5	1806.6	9.8

**Table 3:** Measured 3D error for the six acoustic sources, aggregate statistics, and the mean runtime.

Source	True Position (m)	LS	COM-W	E-COM-W	R-COM-W
1	(36.34,67.55,3.55)	5.380	23.995	10.742	0.783
2	(31.94,57.34,−0.30)	2.450	9.427	1.996	1.076
3	(28.93,45.45,7.30)	5.864	13.263	8.762	0.324
4	(34.61,73.91,−0.40)	21.675	20.792	5.654	1.518
5	(25.85,40.44,−0.20)	2.931	13.478	11.417	1.208
6	(28.61,75.62,−0.40)	2.629	12.724	2.149	2.357
Mean (m)		6.821	15.613	6.787	1.211
Median (m)		4.155	13.371	7.208	1.142
Maximum (m)		21.675	23.995	11.417	2.357
Mean runtime (ms)		12	653	505	19

## Data Availability

The data presented in this study is available on request from the corresponding author.
